# Gut Microbiome Diversity and Composition Correlates With Time in the Therapeutic Range in Patients on Warfarin Treatment: A Pilot Study

**DOI:** 10.1161/ATVBAHA.124.321490

**Published:** 2024-10-31

**Authors:** Pasquale Agosti, Afroditi Kouraki, Tommaso Dionisi, Giovanni Addolorato, Luca D’Innocenzo, Silvia Sorrentino, Flavio De Maio, Erica De Candia, Aitor Blanco-Miguez, Davide Bazzani, Angela Bonadiman, Guendalina Tonidandel, Mattia Bolzan, Francesca Gianniello, Serena M. Passamonti, Maria Abbattista, Ana M. Valdes, Paolo Bucciarelli, Flora Peyvandi, Cristina Menni

**Affiliations:** Department of Pathophysiology and Transplantation, Università Degli Studi di Milano, Italy; Angelo Bianchi Bonomi Hemophilia and Thrombosis Center, Fondazione IRCCS Ca’ Granda Ospedale Maggiore Policlinico, Italy; Academic Unit of Injury, Recovery and Inflammation Sciences, Rheumatology, School of Medicine, University of Nottingham, United Kingdom; NIHR Nottingham Biomedical Research Centre, Nottingham University Hospitals NHS Trust and the University of Nottingham, United Kingdom; Internal Medicine and Alcohol Related Disease Unit, Columbus-Gemelli Hospital; Internal Medicine and Alcohol Related Disease Unit, Columbus-Gemelli Hospital; Fondazione Poli-clinico Universitario A. Gemelli IRCCS, Italy. Department of Medical and Surgical Sciences, Università Cattolica di Roma, Italy; Unit of Hemostasis and Thrombosis; Unit of Hemostasis and Thrombosis; Department of Laboratory and Infectious Sciences; Unit of Hemostasis and Thrombosis; Department of Translational Medicine and Surgery, Università Cattolica del Sacro Cuore, Italy; Pre-Biomics S.r.l., Italy; Pre-Biomics S.r.l., Italy; Pre-Biomics S.r.l., Italy; Pre-Biomics S.r.l., Italy; Pre-Biomics S.r.l., Italy; Angelo Bianchi Bonomi Hemophilia and Thrombosis Center, Fondazione IRCCS Ca’ Granda Ospedale Maggiore Policlinico, Italy; Angelo Bianchi Bonomi Hemophilia and Thrombosis Center, Fondazione IRCCS Ca’ Granda Ospedale Maggiore Policlinico, Italy; Angelo Bianchi Bonomi Hemophilia and Thrombosis Center, Fondazione IRCCS Ca’ Granda Ospedale Maggiore Policlinico, Italy; Academic Unit of Injury, Recovery and Inflammation Sciences, Rheumatology, School of Medicine, University of Nottingham, United Kingdom; NIHR Nottingham Biomedical Research Centre, Nottingham University Hospitals NHS Trust and the University of Nottingham, United Kingdom; Angelo Bianchi Bonomi Hemophilia and Thrombosis Center, Fondazione IRCCS Ca’ Granda Ospedale Maggiore Policlinico, Italy; Department of Pathophysiology and Transplantation, Università Degli Studi di Milano, Italy; Angelo Bianchi Bonomi Hemophilia and Thrombosis Center, Fondazione IRCCS Ca’ Granda Ospedale Maggiore Policlinico, Italy; Department of Pathophysiology and Transplantation, Università Degli Studi di Milano, Italy; Department of Twin Research and Genetic Epidemiology, King’s College London, United Kingdom

**Keywords:** acenocoumarol, gastrointestinal microbiome, metagenome, thrombosis, vitamin K

## Abstract

**T**he proportion of time a patient’s international normalized ratio remains within a therapeutic range (TTR) is critical for the efficacy and safety of vitamin K antagonist anticoagulant therapy (warfarin or acenocumarol).^1^ Poor-quality vitamin K antagonist therapy, as measured by TTR, can cause thrombotic and bleeding complications.^1^ TTR is affected by age, body mass index, genetics, comorbidities, and medications.^1^

The gut microbiota influences the host metabolism and could indirectly affect medication metabolism or anticoagulant absorption, potentially impacting TTR. A recent review^2^ reported that microbial metabolites, vitamin K–producing bacteria, and structural modifications of vitamin K antagonist molecules may indirectly alter vitamin K antagonist medication availability. High taxonomic resolution, functional profiling, and detection of novel microorganisms are possible with shotgun metagenome sequencing.^3^ In this pilot cross-sectional study of warfarin-treated individuals, we examine the relationship between gut metagenome composition and TTR.

## Methods

We enrolled patients on chronic warfarin treatment for any cause for at least 6 months followed at the Gemelli Hospital (Rome). TTR was calculated with the Rosendaal method. Patients with TTR >60% were considered having high TTR (H-TTR, good quality control of anticoagulation) and those with TTR ≤60% as having low TTR (L-TTR, poor quality control of anticoagulation). The research was approved by the Institutional Ethical Committee. All participants provided written informed consent.

### Gut Microbiome Analysis

Collected stool samples were stored at −80° C, within 2 hours of receipt. Deep shotgun metagenomic sequencing (deposited in NCBI bioproject/1165964) was performed by PreBiomics.^3^ MetaPhlAn4 and HUMAnN3.8 were used for taxonomic quantification of the microbial communities at species-level genome bins and to investigate functional capabilities.^3^

### Statistical Analysis

Statistical analysis was performed in R (4.3.2). A multivariable logistic regression, adjusted for age, sex, body mass index, PPI usage, clinical indication for warfarin, and months on warfarin therapy, was used to examine the relationship between H-TTR versus L-TTR and microbiome diversity, identified by the Shannon α-diversity index (as a continuous trait). Results were expressed as odds ratio with 95% CI. The microbiomeMarker package was used to find microbial taxa that explained differences between the 2 groups using linear discriminant analysis (LDA) effect size analysis. We identified significant features using the Wilcoxon rank-sum test and estimated effect sizes via LDA on differentially abundant taxa. LDA effect size was also used to uncover pathways explaining group differences. We selected pathways driven by differentiating genera/species and removed uncommon and housekeeping genes by selecting pathways with median abundance in the top 50% and variance in the top 25%. Taxa and pathways were considered enriched if LDA was >2 and Benjamini-Hochberg *q* values were <0.25.

## Results

The descriptive characteristics of the study population are presented in the Figure (A), corresponding to 40 patients on warfarin treatment: 20 H-TTR and 20 L-TTR.

The Shannon α-diversity index was higher in H-TTR than in L-TTR (odds ratio, 3.85 [95% CI, 1.36–10.87]; *P*=0.01; AUC, 0.84) after adjusting for covariates (Figure [B]).

At the genus level, LDA effect size analysis identified 5 genera enriched in H-TTR, namely *Coprococcus, Barnesiella*, and *Brotolimicola* plus 2 unclassified genera of the families *Barnesiellaceae* and *Eubacteriacaea*. Conversely, the genus *Tyzzerella* was enriched in L-TTR (Figure [C]). At the species level, 16 species were found to discriminate between H-TTR and L-TTR. The 4 top-most discriminative species were *Prevotella marseillensis, Bacteroides stercoris, Barnesiella intestinihominis*, and *Brotolimicola acetigignens*, which were more abundant in H-TTR (Figure [D]).

We investigated microbial pathways associated with TTR and identified a pathway related to folate transformation driven by *B intestinihominis* (upregulated in H-TTR; LDA, 4.03). We also found 9 pathways upregulated in L-TTR driven by 2 *Clostridium* spp. (genus: *Lachnoclostridium*; Figure [E]).

## Discussion

In this small cross-sectional study, we report that gut microbiome composition is linked to TTR in patients anticoagulated with warfarin. H-TTR showed higher gut microbiome diversity, a measure of gut health, compared with L-TTR. We identified several taxa enriched in H-TTR, including (1) *B stercoris*, previously reported to have anti-obesity activity in murine models; (2) *B intestinihominis*, protective against abdominal aortic aneurysm in mice^4^; and (3) *Coprococcus*, an acetate-producing genus. *Tyzzerella*, linked to higher lifetime cardiovascular risk,^5^ was increased in L-TTR.

At the functional level, we found enrichment in folate transformation pathways. The 1-carbon metabolism route, which synthesizes vitamin K biomolecules, is tightly linked to folate, suggesting gut microorganisms may influence vitamin K metabolism through folate pathway interactions. L-TTR boosted dTDP-L-rhamnose production, which many human pathogenic bacteria need for viability and pathogenicity.

Our study benefits from accurate phenotyping and detailed microbiome assessment but is limited by a small sample size, heterogeneity of treatment indication, and possible residual confounders (other medications, comorbidities, diet, and alcohol intake) that were not accounted for.

In conclusion, because improving TTR results in a higher drug efficacy and fewer warfarin-related side effects, and our results suggest that higher microbiome diversity correlates with H-TTR, by targeting the gut microbiome, we may improve anticoagulated patients’ outcomes. Further research is warranted to elucidate the underlying mechanisms and explore the potential therapeutic implications.

## Figures and Tables

**Figure. xxx:**
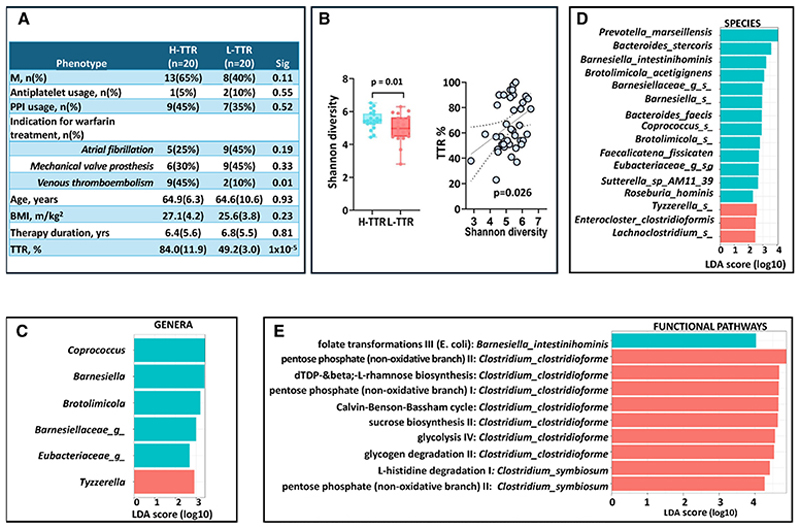
**A**, Descriptive characteristics of the study population comprising 20 middle aged patients (64.9 [SD=5.3] years) with time in the therapeutic range (TTR) >60% (high TTR [H-TTR]) and 20 middle aged patients (64.7 [SD=10.6] years) with TTR ≤60% (low TTR [L-TTR]) enrolled between November 2023 and January 2024. All patients were treated with warfarin following European Society of Cardiology guidelines. Individuals who temporarily interrupted warfarin in the previous 6 months because of surgery or invasive procedures and were switched to heparin, or those who were taking antibiotics were excluded. The 3 main clinical indications for warfarin treatment were (1) prevention of cardioembolic ischemic stroke in atrial fibrillation (n=14); (2) mechanical heart valve prosthesis (n=15); and (3) treatment and secondary prevention of venous thromboembolism (n=11). **B**, Scatterplot depicting the relationship between Shannon alpha diversity and TTR (as a continuous trait) and box plot showing the difference in Shannon alpha diversity in H-TTR (TTR, >60%) and L-TTR (TTR, ≤60%). The difference observed corresponds to 0.91 SD, which has 78% power at alpha=0.05. The Shannon alpha diversity index was calculated using the estimate_richness function from the phyloseq package. **C**, Bar plot showing the linear discriminant analysis (LDA) effect size of the list of genera, (**D**) species and (**E**) pathways enriched in H-TTR and L-TTR using LDA effect size (LEFSe) analysis. We included genera and species with abundance >0.05 present in ≥10% of the sample. The LEFSe analysis indicates differential signatures based on TTR. The LDA scores represent the effect size of each differentially abundant taxa/pathway between H-TTR and L-TTR. It is defined as the degree of consistent difference in abundance between 2 groups and measures how well a feature can differentiate between groups. It is calculated by balancing the feature’s variability and its ability to separate the groups, and then scaling and taking the base-10 logarithm of this value to rank the importance of each feature. Taxa at each level and pathways are shaded blue (H-TTR) or red (L-TTR) in which it is more abundant (*P*<0.01; *q*<0.25; LDA, >2.0). *The model including only covariates (for age, sex, BMI, PPI usage, clinical indication for warfarin, and months on warfarin therapy) achieves an AUC of 0.77. BMI indicates body mass index.

## Data Availability

The data that support the findings of this study are available from the corresponding author upon reasonable request from bonafide researchers. The gut microbiome data are available on NCBI (bioproject number: PRJNA1165964; https://www.ncbi.nlm.nih.gov/bioproject/1165964).

## Nonstandard Abbreviations and Acronyms

H-TTR: high time in the therapeutic range
L-TTR: low time in the therapeutic range
LDA: linear discriminant analysis
TTR: time in the therapeutic range
VKA: vitamin K antagonist
